# Pictorial essay: Neurosurgical application and physics of diffusion tensor imaging with 3D fiber tractography

**DOI:** 10.4103/0971-3026.35818

**Published:** 2008-02

**Authors:** Santosh S Gupta, Zena M Patel, Basant K Misra

**Affiliations:** Departments of Radiology (MRI), P. D. Hinduja Hospital National Hospital and Medical Research Centre, Mahim, Mumbai - 400 016, Maharashtra, India; 1 Departments of Neurosurgery, P. D. Hinduja Hospital National Hospital and Medical Research Centre, Mahim, Mumbai - 400 016, Maharashtra, India

**Keywords:** DTI, MRI, neoplasms, brain

Diffusion tensor imaging (DTI) is a rapidly evolving noninvasive MRI technique for delineating the anatomy and pathology of white matter tracts.[1] This technique, though still mainly a research tool, has been found useful for presurgical planning in patients with intra-axial, focal mass lesions.[2,3] Delineation of the various tracts around a focal lesion and knowledge of the status of infiltration *vs* displacement are vital pieces of information that help neurosurgeons decide on the appropriate surgical approach.

The aim of this article is to provide a pictorial review of DTI with 3D fiber tractography in this context.

## Background and Relevant Physics

The goal of neurosurgery in a focal brain mass lesion is complete removal of the abnormality, while avoiding surgically-induced neurological deficits.[4] Therefore, the proposed margin of surgical dissection should not violate functionally important white matter tracts and eloquent cortical areas. Mapping of these areas is traditionally achieved by various invasive methods which are usually difficult to perform and would by themselves require separate surgical procedures.[5] In these cases, important patient management decisions are often made without a complete knowledge of the anatomic relationships between lesion borders and adjacent, important fiber tracts and the eloquent cortex.

DTI and tractography help in providing at least some part of this information in a noninvasive manner. This technique can also help in the post-operative evaluation of some patients to see the amount of resection and the relationship of the resected margins with different white matter tracts.

Understanding the physics of DTI and tractography involves several complex mathematical calculations and formulae which have been discussed in detail in many review articles.[1,6,7] In brief, diffusion imaging is an MRI imaging technique that is sensitized to the Brownian motion of water molecules in biological tissues.[8] A diffusion-weighted (DW) sequence can be produced by adding a magnetic field gradient of equal magnitude and duration prior to and after the 180-degree refocusing pulse. A routine DW sequence with ADC (apparent diffusion coefficient) provides a measure of the displacement of water molecules in one direction.

**Figure 1:**
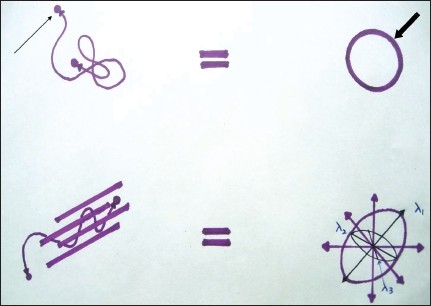
Line diagram demonstrating the concept of isotropic diffusion, which results from the free random movement of protons (arrow) producing a circle (bold arrow). However, in the brain, due to the presence of various fiber tracts, the movement of protons is restricted, resulting in anisotropic diffusion (ellipsoid) (shown in the lower half of the figure)

For DTI, several directions are required, as white matter in the brain is anatomically present in different directions.[9,10] Diffusion in the brain is not uniform but anisotropic, along the direction of the various fiber tracts [Figure 1]. Therefore, the measure of diffusion cannot be represented as a single quantity but is modeled by estimation of a diffusion tensor (D), which is the measurement of water diffusion in different directions. At least six noncollinear directions and an image without diffusion weighting are needed in order to calculate D. From this, the tensor ‘eigen values and eigenvectors’ can be derived.[9,10] The eigenvalues represent the magnitude of diffusion, whereas eigenvectors represent the corresponding direction. The diffusion tensor can be conveniently visualized by a diffusion ellipsoid [Figure 1]. In anisotropic tissues organized in parallel bundles, the largest eigenvalue (λ1) represents the diffusion coefficient along the direction parallel to the fibers, while the two remaining eigenvectors (λ2 and λ3) represent the transverse diffusion coefficients.[9,10]

The magnitude of water diffusion per unit time is described by the term diffusivity[1] and it depends upon impediments imposed on diffusion, regardless of its direction. It is reasonable to speculate that pathologic conditions that alter tissue microstructure would affect not only the bulk diffusivity, but also the anisotropic diffusion characteristics of water and metabolites. The anisotropic part of diffusion in a tissue is measured by fractional anisotropy (FA), which is a rotationally invariant scalar index of the amount of anisotropy. It scales from 0 (no diffusion) to 1 (diffusion in one direction only).[1] Therefore, high FA values represent a high degree of directionality in the white matter tracts compared to grey matter, where the FA values are low. Even in white matter, there are strong variations of anisotropy measures between different brain regions, with the highest measures seen in the corpus callosum and the pyramidal tract. These FA values will alter in any area where there is a focal brain lesion, causing alteration in the white matter tract. This is the principle which was utilized in the application of FA maps in focal brain lesions in our cases.

**Figure 2:**
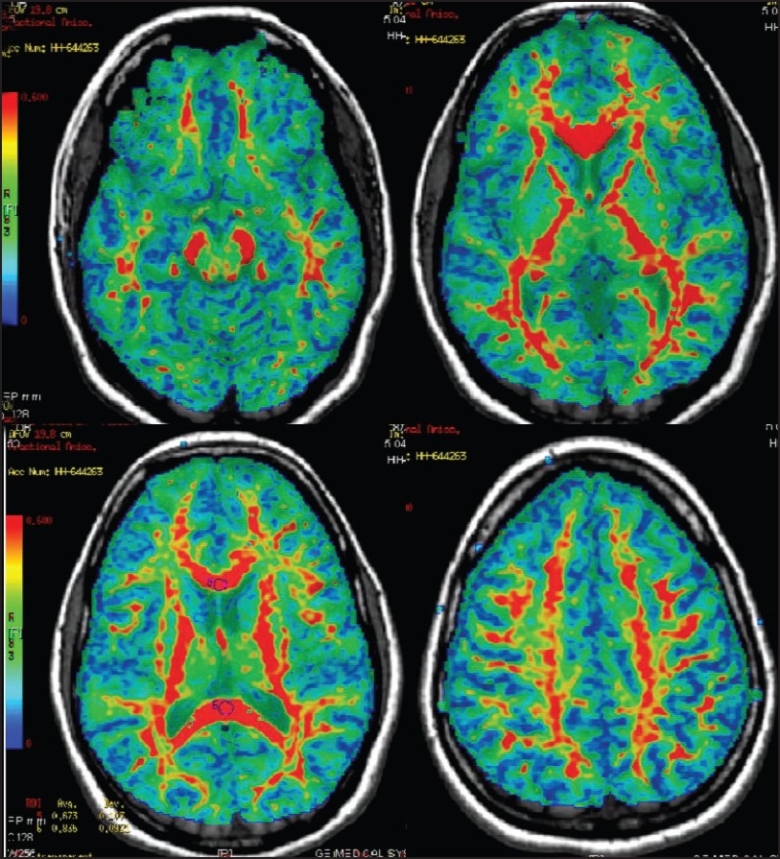
FA maps showing various fiber tracts. Tracts with high FAI value are shown in red, while those with lower FAI values are in blue (note the color scale shown on the right side of the image)

**Figure 3(A-C):**
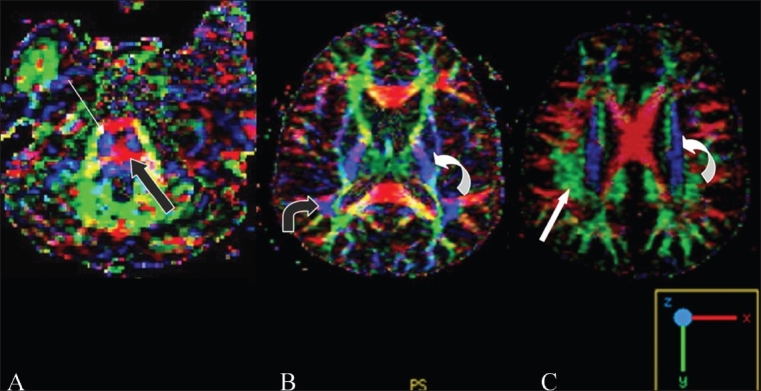
Directionally encoded color maps show the anatomy of the various tracts as per the assigned colors (shown in the lower right corner of the image): at the level of the pons (A), the corticospinal tract (thin white arrow) is seen in blue and the transverse pontine fibers (bold black arrow) are seen in red. Images at higher levels (B, C) show the arcuate fasciculus in blue (bent black arrow), the internal capsule/corona radiata in blue (curved arrows), and the superior longitudinal fasciculus in green (bold white arrow)

Diffusion-encoded FA maps can be acquired according to the scheme proposed by Pajevic and Pierpaoli.[11] In these maps, bright voxels indicate high diffusion anisotropy whereas dark voxels indicate low diffusion anisotropy. On our machine we obtained color FA maps, where the color intensity was scaled in proportion to the magnitude of FA [Figure 2]. From these FA maps, DTI-based color-coded maps can also be generated [Figure 3]. In these maps, colors are chosen according to the high eigenvector associated with the largest eigenvalue. In most MRI machines, red is assigned to the x-direction (left to right), green to the y-direction (anterior-posterior), and blue to the z-direction (superior-inferior).

**Figure 4(A-C):**
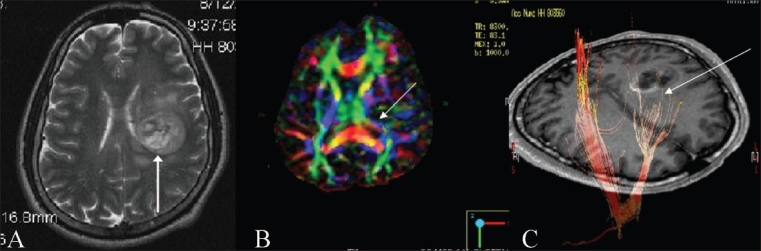
Complete tract disruption. A T2W axial image (A) shows a focal mass lesion (glioblastoma multiforme) (arrow) in the left corona radiata and basal ganglia. Directionally encoded color map shows distortion and altered color in the posterior limb of the left internal capsule (arrow). 3D tractography superimposed on an SPGR sequence shows cut-off of the left corticospinal fibers (arrow), consistent with infiltration. The patient had right sided weakness prior to surgery, which as predicted by DTI, due to infiltration and cut-off of the corticospinal tracts, was not recovered after the surgery.

**Figure 5(A-F):**
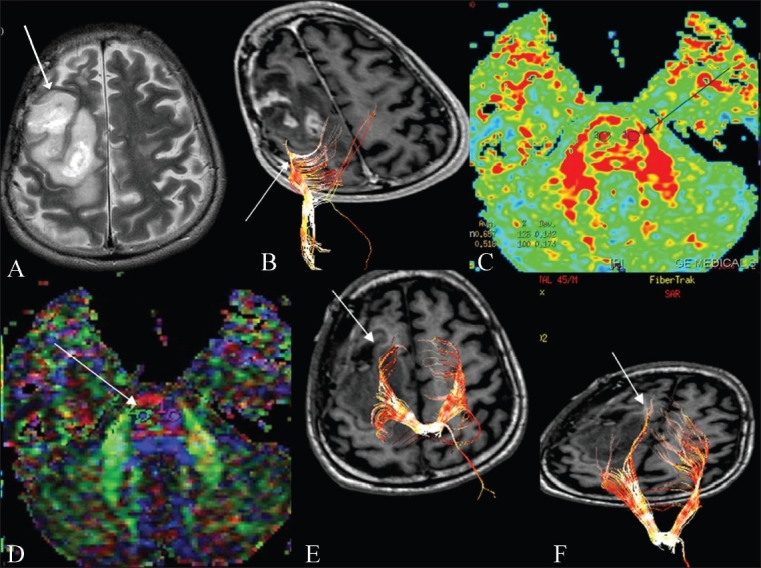
Tract displacement: Pre-operative T2W axial image (A) shows a heterogeneous mass (astrocytoma) (arrow) in the right posterior frontal lobe, reaching the motor cortex posteriorly. 3D tractography image (B) superimposed on a 3D SPGR sequence shows posterior displacement of the corticospinal tract (arrow). The magnified FA image (C) and corresponding directionally encoded color map (D) show how the ROIs (seed) were placed in the region of the corticospinal tracts to obtain the 3D tractography image (B). These images helped the neurosurgeon in taking an anterior approach. Post-operative tractography images (E, F), superimposed on a 3D-SPGR sequence show relocation of the posteriorly displaced corticospinal fibers (arrows), when compared with the pre-operative image (B). The patient had left-sided motor weakness, prior to surgery, which recovered a few days after surgery, as predicted by DTI

By combining the anisotropy data with the directionality it is possible to estimate fiber orientation. This has led to the development of fiber tractography in which three-dimensional pathways of white matter tracts can be reconstructed.[12,13] In this algorithm, called ‘fiber assignment by continuous tracking’ (FACT), reconstruction of the tract is performed by sequentially piecing together discrete and shortly spaced estimates of fiber orientation to form continuous trajectories. Tracking is initiated from the center of a voxel and proceeds according to the direction of the largest eigenvector in that voxel. At the point where the track leaves the voxel and enters the next, its direction is changed to that of the neighbor. In this way, tracking is performed through continuous intercepts between voxels and fiber tract maps are created [Figures 4 and 5].

White matter fibers can be classified into association fibers (which connect two different regions of the cerebral cortex within the same hemisphere, e.g., optic radiation, represented by green); commissural fibers (which connect regions of one hemisphere with those of the opposite, e.g., corpus callosum, represented by red); and projection fibers (which connect the cerebral cortex to the subcortical structures, e.g., corticospinal tract, represented by blue).[7] To see white matter tracts, which connect two different areas of the brain, a seed region of interest (ROI) and a target ROI are used. A small ROI is placed at the site of the desired tract (e.g., an ROI is placed in the anterior pons to evaluate the corticospinal tract) [Figure 5B and C] and the entire tract gets displayed on the 3D tractography image. In the case of the temporal stem, a seed ROI is placed between the superolateral margin of the temporal horn and inferior to the adjacent circular sulcus of the insula.[14]

## Technique

All MRI studies were performed on a standard 1.5 Tesla unit (GE Signa, Gemsow, GE Medical Systems, Milwaukee, WI, USA). A standard quadrature head coil was used. The sequences obtained were axial T2W (TR-4000, TE-95, FOV-22 × 16.5, NEX-2.00, matrix-320 × 320, slice thickness: 5.0/2.0); 3D spoiled-gradient T1W (3D SPGR) (TR-7.8, TE-3.0, FOV-24 × 24, NEX-3.00, matrix-256 × 256, slice thickness: 1.5/0); and diffusion tensor, which consisted of a single, short, spin-echo echoplanar sequence in 25 encoding directions and a diffusion weighting factor of 1000 s/mm^2^. Other parameters were TR-8300, TE-83, FOV-28 × 22, NEX-1.00, matrix-128 × 128, slice thickness: 3.0/0.

Images were post-processed using the GE research software devised for tractography. The maps obtained were (1) FA maps, superimposed on the 3D-SPGR images; (2) directionally-encoded color FA maps; and (3) 3D fiber tractography maps. Anisotropy is maximum in tightly bundled white matter tracts. According to the scale on the left side of the images, higher anisotropy is represented by shades of red and lower anisotropy by blue. Hence, compact white matter tracts are seen in red [Figure 2]. FAI values were plotted on these maps; these were lower in areas involved by the lesion and within edema. The actual direction and anatomy of the tracts is not appreciated on these maps but are seen in the directionally-encoded FA maps, where a specific color is assigned to tracts running in the three orthogonal planes: red is for right to left tracts, green for anteroposterior tracts, and blue for craniocaudal tracts [Figure 3A]. The specific anatomy of the white matter tracts is better seen on these maps [Figure 3B]. A 3D display of tracts can be created [Figure 4C]. For creating 3D fiber tracts, an ROI or seed is placed at the site of the tract on the FA maps [Figure 5B]. For projection fibers, a single seed ROI was sufficient [Figure 5B and C]. However, for association and commissural fibers, a seed ROI was placed at the start and a target ROI at the end of the tract. The entire tract was then obtained in 3D and then superimposed on a T2W image in three planes. Later, these were superimposed on the axial 3D-SPGR image at the level of the lesion and rotated to obtain the best possible views. Sagittal and coronal planes were used when relevant.

## Neurosurgical Application

Our series is based on 20 patients with intra-axial cerebral focal mass lesions. Lesions included were nine astrocytomas, four oligodendrogliomas, two arteriovenous malformations (AVMs), two cavernomas, one cortical dysplasia, one demyelinating disease, and one pyogenic abscess [Table 1].

**Table 1:** Location of focal lesion, with DTI findings and correlation with the clinical pre- and post-operative deficit

Location and histopathology	Pre-op deficit	Tracts infiltrated/displaced or uncertain	Post-op deficit
Lt. frontal OG*	No deficit	CST† displaced, few frontal fibers infiltrated	No deficit
Rt. frontal GBM	Lt. weakness	CST† traversing through the edema. Color-coded map - displaced. FAI uncertain	Improved
Rt.FT† OG*	Lt. weakness	Temporal stem infiltrated, posterior limb of internal capsule displaced on color-coded map, involved on FAI	Persisted
Lt. basal ganglia GBM	Rt. weakness	Posterior limb of internal capsule infiltrated	Persisted
Rt. FT‡ cortical dyspalsia	Lt. paresis	Several frontotemporal tracts involved	Same deficit
Lt. frontal astrocytoma	No deficit	CST† away from lesion, lesion in CSO‖	No deficit
Lt. FP§ tumefactive MS††	Rt weakness	CST† intact	No deficit
Lt TP** AVM	Visual defect	Optic and arcuate fasciculus displaced	Improved
Lt. post-parietal abscess	Rt weakness	Optic radiations displaced, arcuate infiltrated	Improved
Lt. temporal astrocytoma	No deficit	Occipitofrontal fibers infiltrated	No deficit
Lt. FT‡ OG*	No deficit	Corpus callosum displaced, Anterior limb of internal capsule not seen, CST† intact	No deficit
Rt. precental gyrus cavernoma	Lt. upper limb weakness	Motor fibers of CST† displaced anteriorly, not seen post-op	Lt. lower limb weakness
Rt. high parietal AVM	Lt. weakness	Reduced fibers in CST† due to gliosis	Residual deficit
Rt. TP** GBM	No deficit	Optic radiations displaced, posterior fibers of corona radiata and arcuate fasciculus infiltrated	No deficit
Rt. frontal cavernoma	No deficit	CST† intact	No deficit
Lt. TP** OG*	No deficit	TS infiltrated. CST† displaced	No deficit
Rt. FP§ astrocytoma	Lt. weakness	CST† displaced posteriorly	Persisted
Lt. FP§ GBM	Rt. weakness	CST† infiltrated	Improved
Lt. frontal astrocytoma	No deficit	CST† intact, CSO‖ infiltrated	No deficit
Lt. parietal astrocytoma	No deficit	CST† mild anterior displacement	No deficit

* OG Oligodendroglioma,

† CST Corticospinal tract,

‡ FT Frontotemporal,

§ FP Frontpparietal,

‖ CSO Centrum semiovale,

** TP Temporoparietal,

†† MS Multiple sclerosis

**Figure 9(A-D):**
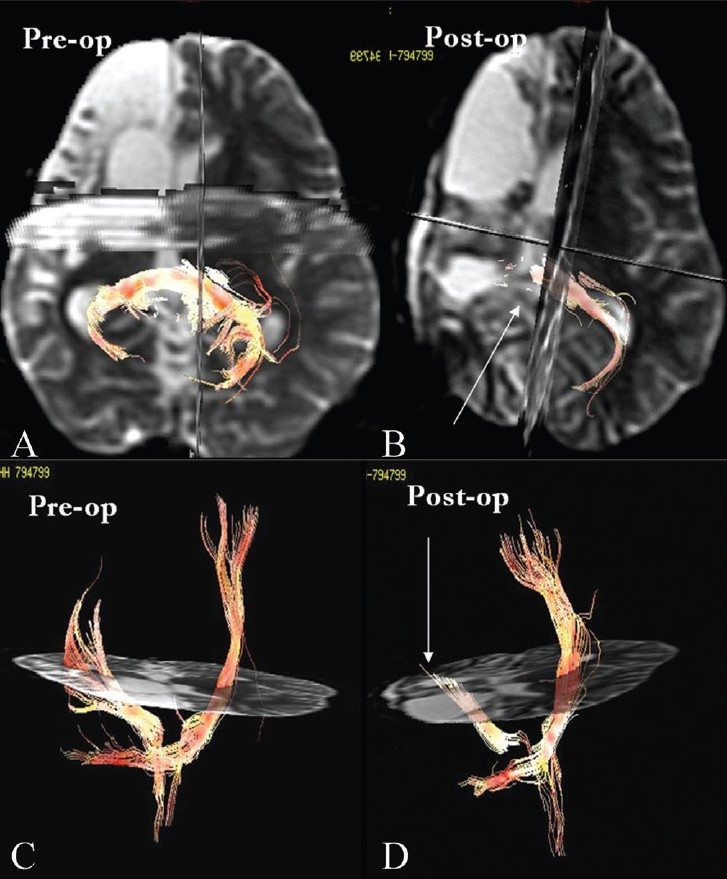
Post-operative tract resection. Pre-operative 3D tractography images (A, C) in a child with right fronto-temporal cortical dysplasia with atrophy, causing left-sided seizures with left hemiparesis, show attenuation of the right corticospinal tract due to marked atrophy. The post-operative images (B, D) after right partial hemispherictomy and callosectomy show clean resection of the corticospinal tracts as well as the corpus callosum (arrows)

Patients were clinically evaluated prior to surgery to look for any neurological deficits, which may occur either due to actual infiltration of the various tracts, mass effect and displacement, or edema. In patients who had just a simple mass effect or edema in a particular tract, it was most logical to assume that they would recover after surgery (provided there was no surgical destruction or damage to the tract, which also can be evaluated on post-operative DTI after comparing with the preoperative data). We used a combination of all the three maps. Various tracts and their outlines, including the colors and orientation, were carefully noted on the DTI study to look for infiltration or simple displacement of the tract. Post-operatively these patients were again clinically evaluated on immediate as well as delayed follow-up to look for any recovery of previously seen neurological deficits or otherwise. In few patients, we also performed post-operative imaging to understand the post-operative anatomy of the tracts [Figure 5] and, in some cases, to see the extent of the resection of the tracts [Figure 9].

The use of DTI and tractography has been described in many neurosurgical cases,[3,15,16] but there have been no randomized trials or outcome studies showing that this technique makes a difference to the final outcome. Newer studies now use a combination of DTI and functional MRI data superimposed on 3D T1W images,[2,15] thereby showing both the white matter tracts and the eloquent cortex.

On the basis of anisotropy and fiber direction or orientation, Field and coworkers have classified the relationship of a tumor to white matter tracts into four categories: deviated, infiltrated, edematous, and destroyed.[17] Our findings were classified into the following categories:

1. Complete tract disruption [Figure 4]
2. Tract displacement [Figures 5 and 6]

**Figure 6(A-C):**
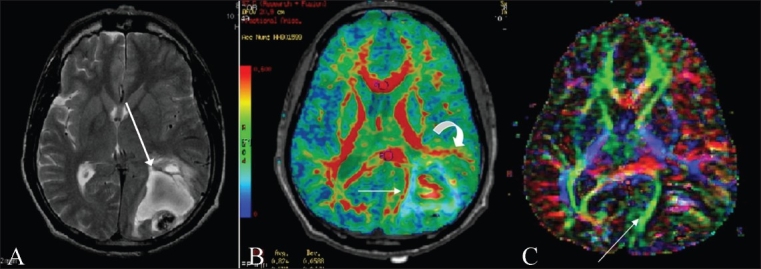
Tract displacement. Left parietooccipital AVM. T2W axial image (A) shows a heterogeneous lesion (arrow) in the left parietooccipital region with flow voids. The FA map (B) shows anterolateral displacement of the arcuate fasciculus (curved arrow) and medial displacement of the optic radiation (straight arrow), also seen on the directionally encoded color map (C) (arrow), with no obvious infiltration of these tracts. This patient had a right-sided visual field defect pre-operatively. As the optic radiation was seen to be only displaced and not infiltrated, the visual loss recovered following surgery, as predicted
3. Tracts traversing through edema [Figure 7].

**Figure 7(A, B):**
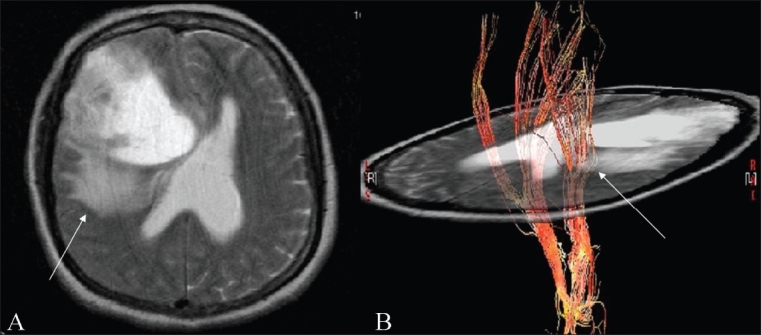
Tracts traversing through edema. DTI in a patient with a large right frontal lobe mass lesion (glioblastoma multiforme) seen on a T2W axial image (A). Note the surrounding hyperintensity/edema (arrow) through which relatively intact corticospinal tract fibers are seen to traverse on the 3D tractography image (B) (arrow), with no evidence of infiltration
4. Temporal stem DTI [Figure 8].

**Figure 8(A-C):**
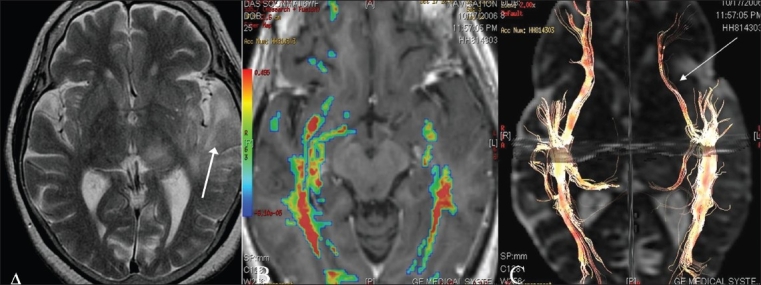
Temporal stem DTI: DTI demonstrating temporal stem anatomy; obtained after placing a seed ROI (not shown) in the region of the temporal stem between the superolateral margin of the temporal horn and inferior to the adjacent circular sulcus of the insula on a coronal image. A T2W axial image (A) shows an ill-defined hyperintense lesion (arrow) (glioma) in the left anterior temporal lobe, extending into the insular cortex. A FAI map (B) superimposed on an SPGR sequence and a 3D tractography image (C) show marked reduction of the left occipito-frontal fasciculus (arrow), suggesting infiltration of these fibers
5. Post-operative tract resection [Figure 9]

Tract disruption/infiltration *vs* displacement were noted in all patients and then correlated with post-operative recovery of the pre-surgical deficit [Table 1].

## Pitfalls and Limitations of DTI

DTI has many pitfalls and a reasonably long learning curve. Technically, it is challenging, especially due to the absence of standardization. There are no proper guidelines for post-processing, with significant variations among different MRI vendors. There is also at present no *in vivo* gold standard for tract visualization with which DTI can be compared.

There are various technical issues as well. Errors in calculating the trajectories can be caused by eddy current distortion, gradient nonlinearities, and motion and susceptibity artifacts.[18,19] There can be problems in mapping of crossing and/or branching fibers, where diffusion anisotropy can average out, causing loss of information or the production of false information about the fiber orientation at that site. There are no standard guidelines as to the threshold to be used, and changing the threshold can significantly alter the number of fibers seen, which can give false information on the 3D tractography images. In our series, the FA values were found to be reduced drastically in areas where the white matter tracts were disrupted and infiltrated by a lesion, as compared to the areas where the tracts were displaced or involved in edema, though the latter finding is not seen in all cases. FA values themselves are not helpful in differentiating tumor infiltration from edema,[17,20] which was also noted in all our cases. Now, newer indices, such as the fiber density index[21] and the tumor infiltration index,[22] have been devised and are being used in order to understand and differentiate tumor infiltration from edema.

Despite these limitations, DTI in neurosurgical cases is a promising clinical tool. With training, experience, and a sound knowledge of its limitations, this technique can offer vital information relevant for planning the surgical approach, specifically in the context of focal brain lesions and the adjacent white matter tracts
